# Altered Functional Connectivity of Fronto-Cingulo-Striatal Circuits during Error Monitoring in Adolescents with a History of Childhood Abuse

**DOI:** 10.3389/fnhum.2018.00007

**Published:** 2018-01-29

**Authors:** Heledd Hart, Lena Lim, Mitul A. Mehta, Charles Curtis, Xiaohui Xu, Gerome Breen, Andrew Simmons, Kah Mirza, Katya Rubia

**Affiliations:** ^1^Department of Child and Adolescent Psychiatry, Institute of Psychiatry, Psychology and Neuroscience, King’s College London, London, United Kingdom; ^2^Lee Kong Chian School of Medicine, Imperial College London-Nanyang Technological University Singapore, London, United Kingdom; ^3^Department of Neuroimaging, Institute of Psychiatry, Psychology and Neuroscience, King’s College London, London, United Kingdom; ^4^MRC SGDP Centre, NIHR BRC for Mental Health, Institute of Psychiatry, Psychology and Neuroscience and SLaM NHS Trust, King’s College London, London, United Kingdom

**Keywords:** functional connectivity, error processing, child abuse, childhood maltreatment, fronto-cingulo-striatal, *MAOA* genotype

## Abstract

Childhood maltreatment is associated with error hypersensitivity. We examined the effect of childhood abuse and abuse-by-gene (*5-HTTLPR, MAOA*) interaction on functional brain connectivity during error processing in medication/drug-free adolescents. Functional connectivity was compared, using generalized psychophysiological interaction (gPPI) analysis of functional magnetic resonance imaging (fMRI) data, between 22 age- and gender-matched medication-naïve and substance abuse-free adolescents exposed to severe childhood abuse and 27 healthy controls, while they performed an individually adjusted tracking stop-signal task, designed to elicit 50% inhibition failures. During inhibition failures, abused participants relative to healthy controls exhibited reduced connectivity between right and left putamen, bilateral caudate and anterior cingulate cortex (ACC), and between right supplementary motor area (SMA) and right inferior and dorsolateral prefrontal cortex. Abuse-related connectivity abnormalities were associated with longer abuse duration. No group differences in connectivity were observed for successful inhibition. The findings suggest that childhood abuse is associated with decreased functional connectivity in fronto-cingulo-striatal networks during error processing. Furthermore that the severity of connectivity abnormalities increases with abuse duration. Reduced connectivity of error detection networks in maltreated individuals may be linked to constant monitoring of errors in order to avoid mistakes which, in abusive contexts, are often associated with harsh punishment.

## Introduction

Childhood maltreatment is, unfortunately, common in the UK, with 22% of 11–17 year olds reporting physical, emotional, sexual abuse or neglect by a caregiver in their lifetime (Radford et al., 2013). Childhood maltreatment is a severe stressor that produces a cascade of physiological, neurochemical and hormonal changes, which can lead to enduring alterations in brain structure, function and connectivity (Teicher et al., 2003) and is associated with many adverse cognitive consequences such as low IQ and academic performance as well as impaired attention, inhibition, emotion and reward processing (Pechtel and Pizzagalli, 2011). Childhood maltreatment is linked to significant volumetric differences, most consistently in lateral and ventromedial fronto-limbic areas and networks (Hart and Rubia, 2012; Lim et al., 2014), but with emerging evidence for alterations also in striatal regions, including caudate and putamen (Cohen et al., 2006; Dannlowski et al., 2012; Teicher, 2015; Frodl et al., 2017).

Cognitive control, particularly the ability to monitor one’s ongoing performance and detect errors, is a key cognitive function critical to mature adaptive behavior (Nachev et al., 2008). Cognitive control and error monitoring deficits have been reported in maltreated (Mezzacappa et al., 2001; Deprince et al., 2009) and institutionalized children (Beckett et al., 2010; Pollak et al., 2010) and in adults who experienced childhood sexual abuse (Navalta et al., 2006). Substantial improvement in cognitive control and error monitoring occurs from childhood to early adulthood, and is underpinned by progressively increasing fronto-cingulo-striatal activation with increasing age during this developmental period (Rubia et al., 2007; Velanova et al., 2008; Rubia, 2013).

Studies of error monitoring have focused mostly on the error-related negativity, an event-related potential (ERP) component associated with error detection localized to the medial frontal/anterior cingulate/supplementary motor area (SMA; Gehring et al., 1993). Enhanced error-related negativity has been associated with early adversity and punitive parental behavior (Meyer et al., 2015) as well as hypervigilance and high sensitivity to punishment (Santesso et al., 2011). Furthermore, increased error-related negativity is typical for psychiatric conditions, such as anxiety and depression, which are commonly associated with childhood maltreatment (Olvet and Hajcak, 2008).

Very few functional magnetic resonance imaging (fMRI) studies of childhood maltreatment have examined error monitoring. Previously published data with this sample by our group suggest that childhood abuse is associated with abnormally increased activation during error monitoring (unsuccessful inhibition trials in a Stop task), compared to controls, in classical dorsomedial frontal error processing regions, in particular the SMA and anterior cingulate cortex (ACC; Lim et al., 2015). Other studies of unsuccessful inhibition in maltreated individuals demonstrate increased activation of posterior and subcortical regions, including the inferior parietal and superior occipital lobe, thalamus, insula, putamen and midbrain (Bruce et al., 2013; Jankowski et al., 2017). It is possible that increased sensitivity of error detection networks observed in maltreated individuals are due to the constant need to monitor their own actions in order to avoid painful mistakes which are often associated with harsh punishment in an abusive context.

Most fMRI studies of childhood maltreatment have concentrated exclusively on functional activation and neglected more sophisticated functional connectivity analyses. Functional communication between brain regions is vital in cognition, thus examination of altered functional connectivity in childhood maltreatment is crucial. fMRI studies of emotion processing demonstrate alterations in limbic–prefrontal connectivity strength (Fonzo et al., 2013; Jedd et al., 2015; Hart et al., in press). Resting state studies report reduced functional connectivity in adults with maltreatment and early life stress histories of diffuse networks including limbic, striatal, frontal, parietal and temporal regions (Bluhm et al., 2009; Van der Werff et al., 2013). There is only one study, to our knowledge, investigating the effect of functional connectivity during response inhibition using the stop task which found that childhood maltreatment is associated with decreased connectivity between inferior frontal cortex (IFC) and ACC despite no change in activation in these regions (Elton et al., 2014). These preliminary findings suggest that it is crucial to better understand the effect of maltreatment on brain networks in addition to isolated regions. This is of particular relevance as childhood trauma has been shown to affect the morphometry and integrity of white matter tracts (Eluvathingal et al., 2006; Paul et al., 2008; Choi et al., 2009) and functional connectivity strength has been shown to correlate with structural connectivity of white matter tracts in the same regions (Damoiseaux and Greicius, 2009).

Although childhood maltreatment is an important risk factor for several psychiatric disorders, it does not invariably lead to dysfunction. It is recognized that genetic differences influence the likelihood that abuse exposure will result in psychopathology (Nugent et al., 2011) so it is important to examine if the abuse-related brain abnormalities are sensitive to gene-by-environment (GxE) interactions. Extensive research supports a role for the brain serotonin system in stress response (Holmes, 2008; El Hage et al., 2009), focusing particularly on the effects of *5-HTTLPR*, a functional polymorphism in the promoter region of the serotonin transporter gene, and the variable number tandem repeat (*VNTR*) functional polymorphism in the promoter region of the monoamine oxidase type A (*MAOA*) gene that selectively metabolizes serotonin, norepinephrine and dopamine (Shih et al., 1999), which are involved in multiple brain functions associated with stress regulation (Charney, 2004). GxE studies on early stress including childhood maltreatment show increased risk for emotional and antisocial behavioral problems in youth with the long (*L*) allele of the *5-HTTLPR* polymorphism (Olsson et al., 2005; Surtees et al., 2006; Chipman et al., 2007; Chorbov et al., 2007; Laucht et al., 2009) and with the low activity variant of the *MAOA-uVNTR* polymorphism (Caspi et al., 2002; Kim-Cohen et al., 2006; Cicchetti et al., 2007; Taylor and Kim-Cohen, 2007; Weder et al., 2009). The *MAOA-Low* allele is also associated with changes in orbitofrontal volume, amygdala and hippocampus hyperreactivity during aversive recall, and impaired cingulate activation during cognitive inhibition (Meyer-Lindenberg et al., 2006). The size of the current sample is very small for conducting genotype analyses, and the analyses are hence underpowered. Nevertheless, given that we test a very specific hypothesis of an association with a few *a priori* selected genotypes on specific neural networks, we considered it informative to add this explorative analysis that could be useful for future studies.

This study therefore examined the association between severe childhood maltreatment and functional connectivity of error processing networks in medication-naïve, drug-free young people using a challenging tracking stop task which ensures 50% inhibition failures and is hence optimally suited to test error detection networks. Although we found no brain function abnormalities in this group of abused adolescents relative to controls during successful stop trials, given the findings of Elton of functional connectivity deficits in adults with childhood maltreatment histories despite no differences in inhibition-related activation, we also tested connectivity deficits during inhibition. Functional connectivity of all seed regions was therefore analyzed for both error processing and inhibition. Sexual abuse was excluded due to the known differences in structural, behavioral and psychiatric consequences (Ackerman et al., 1998; Heim et al., 2013). Based on evidence of the role of fronto-cingulo-striatal regions in error monitoring (Rubia et al., 2007), and altered structure and function of these regions in individuals with a history of childhood maltreatment (Carrion et al., 2008; Dannlowski et al., 2012; Hart and Rubia, 2012; Frodl et al., 2017), and in particular our previous findings in this sample of increased ACC activation during error monitoring (Lim et al., 2015), we hypothesized that the abused group, relative to healthy controls, would have decreased functional connectivity of dorsomedial fronto-cingulo-striatal networks, particularly prefrontal cortex, ACC, caudate and putamen, during error monitoring. We also explored if these abnormalities would be moderated by *5-HTTLPR* and *MAOA* polymorphisms in this limited sample.

## Materials and Methods

### Participants

Fifty right-handed adolescents aged 13–20 years participated (Table 1). Twenty-three physically maltreated participants were recruited through the former London charity Kids Company, Child and Adolescent Mental Health Services (CAMHS) and advertisements. Maltreated participants had experienced severe physical abuse prior to age 12, as defined by scores of ≥13 on the physical abuse subscale of the Childhood Trauma Questionnaire (CTQ; Bernstein and Fink, 1998). Participants who scored ≥13 for physical abuse were interviewed using the Childhood Experience of Care and Abuse (CECA) Interview (Bifulco et al., 1994) to ascertain more detailed information. Information regarding maltreatment histories was corroborated (with consent) from social services. Physically abused participants frequently had also experienced concurrent emotional abuse, emotional neglect or physical neglect (Table 1) and were assessed using the Development and Well Being Assessment (DAWBA; Goodman et al., 2000) with psychiatric diagnoses being assigned by an experienced child psychiatrist (KM).

**Table 1:** Demographic characteristics of 22 young people exposed to severe childhood abuse and 27 healthy controls.

	Childhood maltreatment (*N* = 22)		Healthy controls (*N* = 27)	
	Mean	SD	Mean	SD
**Age (years)**	17.2	2.44	17.5	1.63
**Socioeconomic status**	2.77	0.69	3.22	0.75
**IQ**	91.7	15.2	105.4	10.1
**Childhood trauma questionnaire**:				
*Physical abuse*	21	4.16	5.52	0.94
*Emotional abuse*	17.8	4.21	6.04	1.13
*Sexual abuse*	5.14	0.66	5.11	0.42
*Physical neglect*	13.8	5.23	5.59	1.22
*Emotional neglect*	17.9	4.74	7.93	3.35
*Total CTQ score*	74.14	16.72	30.81	11.78
**Age at onset of (physical) abuse (years)**	4.05	2.73		
**Duration of (physical) abuse (years)**	8.27	3.12		
	*N*	**%**	*N*	**%**
**Gender (Males)**	15	68	21	77
**Ethnicity:**				
*Caucasian*	10	45	13	48
*Afro-Caribbean*	9	41	12	44
*Others (Asian/mixed)*	3	14	2	8
**Psychiatric diagnosis:**				
*None*	3	14	27	100
*PTSD*	13	59	−	
*Depression*	6	27	−	
*Anxiety disorders*	5	23	−	
*Social phobia*	1	5	−	
*ADHD*	1	5		
*ODD/CD/Other disruptive behaviors*	5	23	−	

*CA, Childhood Abuse; HC, Healthy Controls; ADHD, Attention Deficit Hyperactivity Disorder; PTSD, Post-Traumatic Stress Disorder; ODD, Oppositional Defiant Disorder; CD, Conduct Disorder*.

Twenty seven healthy controls were recruited from advertisements, had experienced no maltreatment (CTQ subscale scores of ≤7 for physical abuse, ≤8 for emotional abuse, ≤6 for sexual abuse, ≤9 for emotional neglect and ≤7 for physical neglect) and had no psychiatric diagnoses (again assessed by KM using the DAWBA). The control group was matched as closely as possible to the maltreated group in ethnicity and gender.

In addition to the CTQ and DAWBA, all participants underwent the Wechsler Abbreviated Scale of Intelligence (WASI; Wechsler, 1999) to assess IQ. Socioeconomic status (SES) was measured by two items from the Family Affluence Scale (FAS; Currie et al., 2008) on housing tenure and room occupancy. A 10 panel T-cup urine test^1^ was used to test for substance abuse and participants who tested positive for any of the 10 substances were excluded from taking part. Other exclusion criteria were left-handedness, IQ < 70, current psychoactive medication, sexual abuse (as defined by a score of ≥6 on the sexual abuse subscale of the CTQ), neurological disorder, major head injuries, drug and alcohol abuse, literacy problems, learning disability, psychotic illness, bipolar disorder, schizophrenia, current suicidal behavior or general MRI contraindications. Participants received £40 to compensate for their time and travel. The National Research Ethics Service Committee London, Fulham, reviewed and approved the study (reference 11/LO/0799) and informed written consent was obtained from all participants and, if below 18 years old, informed written consent was also obtained from parents or guardians.

### Genotyping

The *5-HTTLPR* promoter region polymorphism (44 base pair insertion/deletion) was genotyped using the methods described in an earlier study (Zoroğlu et al., 2002). The alleles were designated S (484 bp) and L (528 bp). The *MAOA* 30 bp-promoter *uVNTR* was genotyped using the method described in an earlier study (Deckert et al., 1999). The alleles of the *MAOA*-*uVNTR* were grouped into two classes (short allele: 2, 3; long allele: 3.5, 4) for the analysis based on the functional roles that enzyme expression is relatively high for the long allele (*MAOA-High*) and lower for carriers of the short allele *(MAOA-Low)*.

### fMRI Paradigm: Stop Task

**Figure 1:**
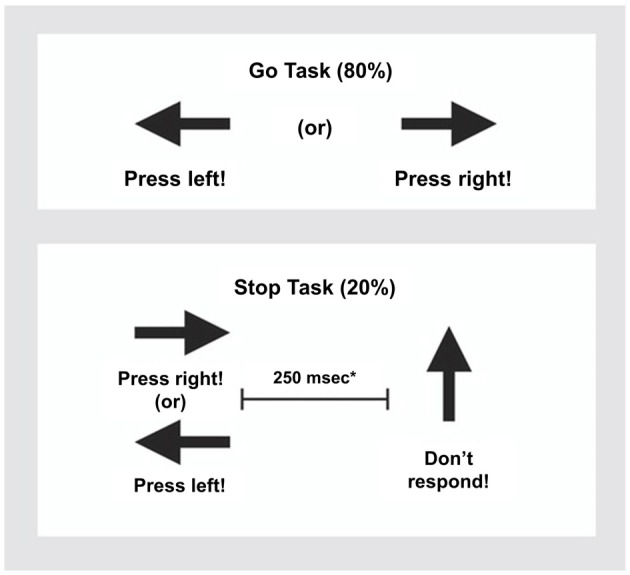
Schematic presentation of the tracking Stop Task. Subjects have to respond to go arrows that point either right or left with a right/left button response. In 20% of trials, the go signals are followed (about 250 ms later) by stop signals and subjects had to inhibit their motor responses. *A tracking algorithm changes the time interval between go signals and stop signals according to each subject’s performance on previous trials (average percentage of inhibition over previous stop trials, recalculated after each stop trial), resulting in 50% successful and 50% unsuccessful inhibition trials.

The rapid, mixed trial, event-related fMRI design was practiced by participants once before scanning. The visual tracking stop task requires withholding a motor response to a go stimulus when it is followed unpredictably by a stop signal (Rubia et al., 2003, 2007, 2013). The basic task is a choice reaction time task where subjects have to respond as fast as they can with their right or left index finger to go signals, which consists of left and right pointing arrows. The mean inter-stimulus interval is 1.8 s (234 go trials). In 20% of trials, pseudo-randomly interspersed, the go signals are followed (about 250 ms later) by arrows pointing upwards (stop signals), and participants have to inhibit their motor responses to these trials (60 stop trials). A tracking algorithm changes the time interval between go-signal and stop-signal onsets according to each participant’s inhibitory performance. The interval increases in steps of 50 ms if the participant’s percentage of inhibition is above 50%, making it more difficult to inhibit and decreases in steps of 50 ms if the participants performance is below 50%, making it easier to inhibit to the stop signals. This tracking algorithm ensures that the task is equally challenging for everyone as participants work at the edge of their own inhibitory capacity and provides 50% successful and 50% unsuccessful inhibition trials at every moment of the task (Figure 1, see Rubia et al., 2003 for further task details). This allows us to analyze an equal number of failed and successful stop trials. In the fMRI analysis, brain activation to the failed and successful stop trials is contrasted with the implicit baseline go trials (i.e., failed stop—go trials; successful stop-go trials).

### Performance Data Analysis

Independent sample *t*-tests were used to compare the main variables of the stop task performance between the abused and the control group using SPSS 21: stop-signal reaction time, mean reaction time to go trials, post-error reaction time, omission errors and the probability of inhibition to stop trials.

### fMRI Image Acquisition

Gradient-echo echoplanar MR imaging (EPI) data were acquired on a GE SIGNA HDx 3T system at the Centre for Neuroimaging Sciences, King’s College London. A semi-automated quality control procedure ensured consistent image quality (Simmons et al., 1999). The body coil was used for RF transmission and an eight channel headcoil for reception. In each of 23 non-contiguous planes parallel to the anterior-posterior commissure, 237 interleaved T2*-weighted MR images depicting BOLD (Blood Oxygen Level Dependent) contrast covering the whole brain were acquired with TE = 40 ms, TR = 2 s, flip angle = 75°, in-plane resolution = 3 mm, slice thickness = 5 mm (slice-skip = 0.5 mm). A high-resolution gradient EPI was also acquired in the inter-commissural plane, with TE = 30 ms, TR = 1.8 s, flip angle = 90°, 43 slices, slice thickness = 3.0 mm, slice skip = 0.3 mm, 1.875 mm in-plane voxel size (matrix size 128 × 128), providing complete brain coverage.

### fMRI Data Analysis

#### Movement

Head motion is a well-known confound of both resting state functional connectivity (Power et al., 2012; Van Dijk et al., 2012) and task based fMRI data (Siegel et al., 2014). In order to reduce the likelihood of false positives caused by head movement we therefore excluded participants with root mean square (RMS) realignment estimates exceeding 1 mm. This was calculated from realignment parameters (rotational estimates converted to translational at radius of 50 mm) as described by Siegel et al. (2014) and resulted in the exclusion of one maltreated participant, leaving a final sample of 22 participants in the childhood abuse group. All healthy controls had RMS movement <1 mm. Multivariate analyses of variance (MANOVAs) were used to test for between group differences in the extent of 3-dimensional motion as measured by values for maximum and mean displacement for x, y and z axes.

#### Preprocessing

Data were analyzed using SPM8^2^. Images were motion corrected with all images being realigned to the first scan in the time-series and then the mean image. After realignment, images were co-registered to the high resolution EPI. All scans were normalized to standard space, using the EPI template, with the parameters derived from the high resolution EPI and applied to the functional time series. Data were spatially smoothed using a kernel of 8 mm full-width half-maximum.

#### Functional Activation Analyses

After preprocessing, data were analyzed within the framework of the general linear model. A first-level model was created for each participant, including regressors encoding unsuccessful stop and successful stop trials. Movement parameters from the realignment procedure were included in the model as regressors of no interest. For second-level (group) analyses, contrast images from the first-level analysis were used to conduct full factorial whole-brain analyses for each condition. These results are published elsewhere (Lim et al., 2015).

### Functional Connectivity Analysis

To assess differences in functional connectivity between groups during error processing and motor response inhibition, a generalized psychophysiological interaction (gPPI) analysis was conducted using SPM8. Ten seed regions were selected based on information obtained from prior neuroimaging studies of error monitoring and motor response inhibition. (1) Left ACC (−6, 38, 16); (2) right ACC (6, 38, 16); (3) left caudate (−10, 18, 4); (4) right caudate (11, 17, 4); (5) left IFC (−47, 31, 13); (6) right IFC (49, 31, 13); 3); (7) left putamen (−22, 12, 1); (8) right putamen (25, 12, 0); (9) left SMA (−10, 0, 62); and (10) right SMA (6, 0, 62). These seed regions were chosen based on widespread evidence for their involvement in error processing and motor response inhibition in children and adults (Rubia et al., 2001, 2003, 2011, 2013; Simmonds et al., 2008; Swick et al., 2008; Aron et al., 2014; Hart et al., 2014). Co-ordinates for all seed regions were selected as the centroids of the region of interest (ROI) as defined using wfupickatlas (Maldjian et al., 2003) and aal (Tzourio-Mazoyer et al., 2002). For each seed region, at the individual subject level, an average time course was extracted defined as an 8 mm sphere around the abovementioned coordinates for use in the gPPI analysis.

We carried out the functional connectivity analysis using the gPPI toolbox^3^. Compared with standard PPI implementation in SPM, gPPI allows for interaction of more than two task conditions in the same PPI model and improves model fit, specificity to true negative findings, and sensitivity to true positive findings (McLaren et al., 2012). Here, we investigated the gPPI (interaction effect) during our contrasts of interest unsuccessful stop vs. go (error) and successful stop vs. go (inhibition) for all 10 seed regions. Thus we extracted the mean time series for each participant from the 10 seed regions and analyzed functional connectivity differences during both error processing and response inhibition. For each participant, the gPPI analysis was performed on the first level separately for each seed region and included the categorical regressors for unsuccessful and successful stop conditions. The deconvolved time series from the seed region was extracted for each participant to create the physiological variable. The condition onset times were separately convolved with the canonical hemeodynamic response function for each condition, creating the psychological regressors. The interaction terms (PPIs) were computed by multiplying the time series from the psychological regressors with the physiological variable. To examine the effect of the interaction terms, activity within the seed region was regressed on a voxel wise basis against the interaction, with the physiological and psychological variables serving as regressors of interest. The individual gPPI contrast images were entered into separate second level analyses to compare groups. Thus, the resulting activation maps from this analysis correspond to group differences for functional connectivity between the seed region and other brain regions during: (1) error processing and (2) inhibition. Results are reported using a cluster threshold of *p* < 0.05 family-wise error rate (FWER) corrected. Given the limited studies testing brain function differences in physically abused populations, and to control for the false positive rate (using *p* < 0.05 FWER random field theory-corrected cluster statistics) while limiting potential type II errors, we chose an* a priori* cluster-forming threshold of *P* < 0.001 for significant between-group differences, with an extent threshold of 10 voxels.

Finally, significant clusters were extracted for exploratory correlational analysis with the abuse measures (onset, duration, CTQ score) within the maltreated group. Preliminary analysis of GxE effect on the significant clusters was conducted using ANOVAs with group and genotype (*5-HTTLPR, MAOA)* as between-subject factors.

## Results

### Subject Characteristics

Groups did not differ significantly on age (*t*_(47)_ = 0.46, *p* = 0.65), gender (*t*_(47)_ = 0.16, *p* = 0.87), ethnicity (*t*_(47)_ = 0.58, *p* = 0.58) nor SES (*t*_(47)_ = 1.24, *p* = 0.22) but differed on IQ as expected (*t*_(47)_ = 3.76, *p* < 0.001; Table 1). Since lower IQ is associated with childhood maltreatment (De Bellis et al., 2009), artificially matching groups on IQ is inappropriate as it creates unrepresentative groups; either the abused group will have higher IQs than the abused population or the control group will have IQs below normative expectations (Dennis et al., 2009). Also, it is misguided to control for IQ differences by covarying IQ when groups are not randomly selected and the covariate is a pre-existing group difference as ANCOVA would lead to potentially spurious results (Miller and Chapman, 2001; Dennis et al., 2009). The data are therefore presented without matching or covarying IQ. However, to explore and rule out any potential influence of IQ, an analysis of covariance (ANCOVA) covarying for IQ was conducted.

Although we selected participants with severe childhood physical abuse, they also experienced marked/severe childhood emotional abuse and neglect (Table 1) which typically co-occur with physical abuse, and hence are a representative group of the abused population (Edwards et al., 2003; Trickett et al., 2011).

### Task Performance

Mean performance values are reported in Table 2. As expected, the probability of inhibition was about 50% in all participants with no significant group differences, showing that the task algorithm was successful (*t*_(47)_ = 1.04; *p* = 0.31).

**Table 2:** Stop task performance of 22 young people exposed to severe childhood abuse and 27 healthy controls.

	Childhood maltreatment (*N* = 22)		Healthy controls (*N* = 27)	
Performance measure	Mean	SD	Mean	SD
Stop signal reaction time (ms)*	132	158	117	106
Stop signal delay	425	180	370	150
Go signal reaction time (ms)	557	97	487	87
Post-error reaction time (ms)	576	129	487	97
Probability of inhibition (%)	52	7	50	3
Omission errors to go signals	16	25	5	11

CA, Childhood Abuse; HC, Healthy Controls. *Calculated by subtracting the mean stop signal delay (the average time between go and stop signal, at which the participant managed to inhibit to 50% of trials) from the mean reaction time to go trial.

Groups differed significantly on mean reaction time to go trials (*t*_(47)_ = 2.68, *p* < 0.02) and post-error reaction time to go trials (*t*_(47)_ = 2.76, *p* < 0.009) but not on stop-signal reaction time (*t*_(47)_ = 0.37, *p* = 0.7). Abused participants were significantly slower in their mean reaction time and post-error reaction time than healthy controls, suggesting they were slower in response to go trials and more cautious in go trials after they made a mistake (*p* < 0.05).

### Functional Brain Activation Analyses

#### Movement

MANOVAs showed no significant group effects in the extent of 3-dimensional motion as measured by maximum (*F*_(3,45)_ = 2.10; *p* = 0.12) and mean (*F*_(3,45)_ = 0.91; *p* = 0.346) displacement for x, y and z axes.

#### Functional Activation

Within and between group functional brain activation is reported elsewhere (Lim et al., 2015). The main finding was that, during unsuccessful inhibition, the childhood abuse group showed increased brain activation relative to the healthy comparison group in typical error processing regions of the dorsomedial frontal cortex, including bilateral SMA and ACC.

### Functional Connectivity

#### Within Group Connectivity Maps

**Figure 2:**
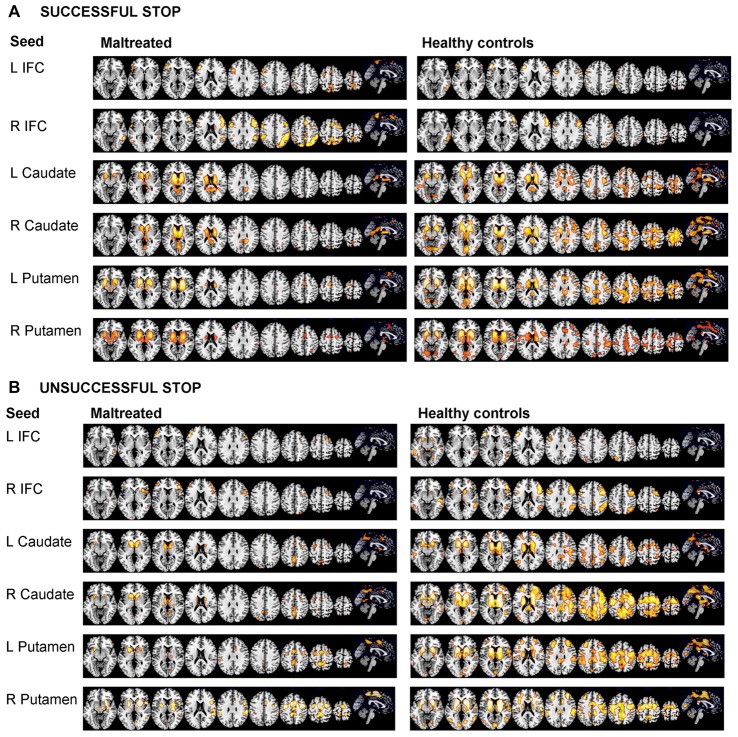
Within group functional connectivity for the 10 seed regions for **(A)** Response Inhibition and **(B)** Error Processing. The threshold is *P* < 0.05 FWE corrected. The right of the image corresponds to the right side of the brain. L, left; R, right; ACC, anterior cingulate cortex; IFC, inferior frontal cortex; SMA, supplementary motor area.

Figures 2A,B show within group functional connectivity maps for the different seed regions for stop errors and successful stop trials, respectively.

### Between Group Functional Connectivity Differences

**Figure 3:**
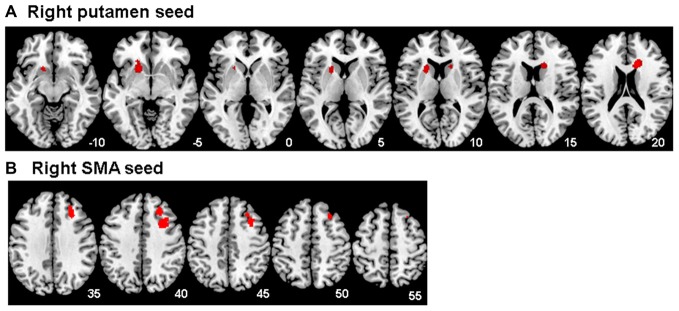
Between group differences in functional connectivity for Maltreated < healthy controls for **(A)** the right putamen seed region and **(B)** the right supplementary motor area (SMA) seed region for the unsuccessful stop vs. go contrast. The threshold is *P* < 0.05 FWE corrected at the cluster level. Z-coordinates represent distance from the anterior–posterior commissure in mm. The right side of the image corresponds to the right side of the brain.

A significant group effect for connectivity was revealed for the right putamen seed region with bilateral caudate, right anterior cingulate cortex and left putamen during unsuccessful stop vs. go (*F*_(1,47)_ = 19.01; *p* < 0.001). *Post hoc* comparisons showed that, during error processing only, maltreated adolescents relative to healthy controls had reduced connectivity between right putamen and bilateral caudate, right anterior cingulate cortex and left putamen (Table 3, Figure 3). For the right SMA seed region, a significant group effect for functional connectivity was revealed with right middle/superior frontal gyrus (MFG/SFG), including dorsomedial and dorsolateral prefrontal cortex (DMPFC/DLPFC), and inferior frontal gyrus (IFG; BA 8/9/44) for unsuccessful stop vs. go (*F*_(1,47)_ = 18.55; *p* < 0.001). This was due to reduced connectivity for maltreated adolescents compared to controls between right SMA and right DMPFC, DLPFC and IFG during error processing (Table 3, Figure 3). At the FWER corrected cluster threshold of *p* < 0.05 no effect of group was observed for the remaining eight seed regions during unsuccessful inhibition, nor for any of the 10 seed regions during successful inhibition.

**Table 3:** Regions demonstrating differential functional connectivity with the right putamen and supplementary motor area (SMA) seed regions during unsuccessful stop vs. go response trials for 22 young people exposed to severe childhood abuse and 27 healthy controls.

		Cluster level		Peak	Voxel level
Seed region	Comparison and brain region	No. of voxels	*p (corr.)*	MNI coordinates	*Z*
	**Childhood maltreatment < healthy controls**
**Right putamen**	Right caudate and anterior cingulate cortex (BA32)	171	0.015	16, 22, 20	4.55	
	Left caudate and putamen	211	0.012	−16, 16, −8	4.06	
**Right SMA**	Right dorsomedial prefrontal cortex, dorsolateral prefrontal cortex, inferior frontal gyrus (BA 8/9/44)	423	0.034	26, 32, 38	3.93	

*P-value is < 0.05 FWER corrected*.

### Exploratory Analyses

#### Correlational Analysis

The significant clusters were extracted and correlated with abuse measures (severity/CTQ score, age of onset and duration of abuse), controlling for IQ, age and gender, within the abuse group only. Longer abuse duration was associated with reduced functional connectivity between right putamen and left caudate and putamen (*r* = 0.52, *p* < 0.05) and between right SMA and right DMPFC (*r* = 0.46, *p* < 0.05). No other significant correlations were found. A correlation matrix was generated to investigate relationships for the maltreated group between gPPI values and task performance, movement, IQ, age, gender and clinical symptom measures (see Table 4). The only unexpected significant correlation was between right SMA/DMPFC connectivity and gender, with females generally having lower gPPI values and therefore being more associated with reduced functional connectivity between right SMA and right DMPFC than males. No correlations were observed between gPPI values and movement, IQ, age nor performance measures.

**Table 4:** A correlation matrix showing Pearson correlation coefficients for the maltreated group only for generalized psychophysiological interaction (gPPI) values for left caudate and putamen connectivity (with right putamen seed), gPPI values for right DMPFC (with right SMA seed), age, IQ, gender, clinical symptom measurements, task performance measures and movement (mean displacement).

	L Caudate/Putamen	R DMPFC	Age	IQ	Gender	Clinical	Go RT	Post error RT	Stop signal RT	Mean displacement
L Caudate/Putamen	1	0.419	−0.121	0.320	−0.103	−0.223	−0.227	−0.260	0.148	−0.057
R DMPFC	0.419	1	−0.302	0.187	0.444*	0.419	−0.002	−0.002	0.211	0.097
Age	−0.121	−0.302	1	−0.271	−0.257	−0.291	−0.172	−0.182	−0.314	0.243
IQ	0.320	0.187	−0.271	1	−0.080	0.193	−0.167	−0.160	0.064	−0.277
Gender	−0.103	0.444*	−0.257	−0.080	1	0.034	0.349	0.309	0.300	−0.297
Clinical	−0.223	0.419	−0.291	0.193	0.034	1	0.138	0.125	−0.020	−0.357
Go RT	−0.227	−0.002	−0.172	−0.167	0.349	0.138	1	0.995**	0.065	−0.166
Post error RT	−0.260	−0.002	−0.182	−0.160	0.309	0.125	0.995**	1	0.043	−0.132
Stop signal RT	0.148	0.211	−0.314	0.064	0.300	−0.020	0.065	0.043	1	−0.223
Mean displacement	−0.057	0.097	0.243	−0.277	−0.297	−0.357	−0.166	−0.132	−0.223	1

*Correlation is significant at the 0.05 level (2-tailed); **Correlation is significant at the 0.01 level (2-tailed). DMPFC, dorsomedial prefrontal cortex; RT, reaction time; SMA, supplementary motor area.

#### IQ ANCOVA Analysis

Given that the maltreated group had a significantly lower mean IQ than the healthy comparison group, data were reanalyzed covarying for IQ. All main findings remained significant.

#### IQ Subset Comparison

The healthy control participants with the five highest IQ values were excluded and functional connectivity analyses were repeated. We found that the effect size for the significant connectivity abnormality finding between the right putamen seed region and bilateral caudate, right anterior cingulate cortex and left putamen for maltreated adolescents compared to healthy adolescents, was Cohen’s *d* = 1.06 for the original comparison and *d* = 1.02 for the subset excluding the controls with highest IQ. To establish whether the group differences between the whole sample comparison to controls and the subset comparison to controls were significantly different, we directly compared the effect sizes of the group differences (Matthews and Altman, 1996) using the z-test. The difference between the two effect sizes (es) can be considered a normalized variable, where the standard error (se) of the difference is a combination of the standard errors of the two comparisons. Based upon this, the probability of a Type I error can be calculated using the formula: *p*(α) = (es_1_ − es_2_)/sqrt(se_1_^2^ + se_2_^2^). The Effect sizes did not significantly differ (*z* = 0.87; *p* = 0.38).

#### GxE Analysis

**Figure 4:**
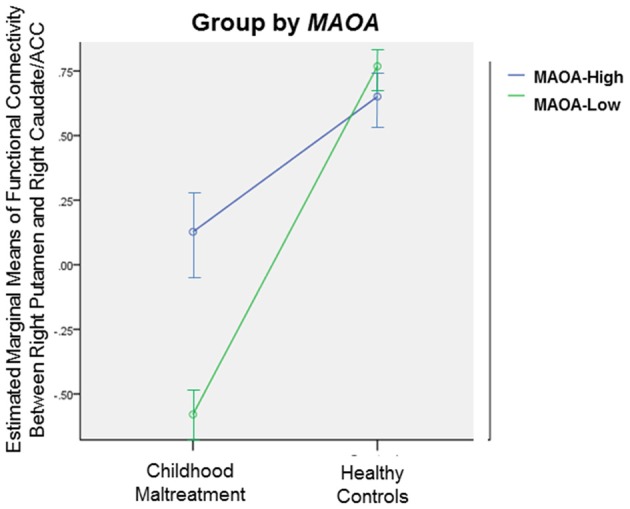
Significant gene-by-environment (GxE) interaction effect between group (childhood abuse vs. healthy controls) and monoamine oxidase type A (MAOA) genotype (MAOA-Low vs. MAOA-High) on functional connectivity between right Putamen and right Caudate/ACC, *p* < 0.05. Error bars represent 95% confidence interval.

Exploratory GxE analysis was conducted on the brain regions that differed in connectivity between the abuse group and healthy controls. ANOVAs with group (maltreated vs. healthy controls) and each genotype (5-*HTTLPR LL* vs. *S* carriers; *MAOA-Low* vs.* MAOA-High*) as between-subject factors were conducted. There was a significant group-by-*MAOA* effect on connectivity between right putamen and right caudate and ACC (*F*_(1,43)_ = 4.57, *p* < 0.05), due to a greater deficit in *MAOA-Low* individuals exposed to abuse than *MAOA-Low* healthy individuals (Figure 4). No significant group-by-5-*HTTLPR* effect was observed.

## Discussion

To our knowledge, this is the first study examining the association between severe childhood abuse and functional connectivity of brain networks during error monitoring in medication-naïve, drug-free young people. Furthermore, it is the first study to explore GxE effects on maltreatment-related connectivity abnormalities. Behaviorally, maltreated individuals had slower go and post-error reaction times than healthy controls, but no abnormalities in the inhibition measure. Abused participants relative to healthy controls exhibited significantly reduced functional connectivity between right putamen and left putamen, bilateral caudate, and right ACC and between right SMA and IFC and DLPFC/DMPFC during error monitoring. Reduced connectivity between right putamen and left caudate and putamen and between right SMA and MFG was associated with longer abuse duration. Exploratory analyses suggest that abuse-related deficits in right hemispheric putamen-caudate-ACC connectivity may be moderated by *MAOA* genotype. No significant group differences in functional connectivity were observed during successful inhibition, suggesting that the abnormalities were specific to error processing.

ACC, SMA, DMPFC and IFG are typical regions involved in error processing and performance monitoring in healthy individuals on the same or similar fMRI stop paradigms and, whilst the basal ganglia, including putamen and caudate, are more typically involved in response inhibition, there is also evidence that they may play a role in error processing (Rubia et al., 2003, 2007, 2011, 2013; Li et al., 2008; Stevens et al., 2009; Rubia, 2013; Hochman et al., 2015). The structure and function of the ACC and frontal cortices, including DLPFC, MFG and IFG, are consistently reported to be affected by childhood maltreatment (Hart and Rubia, 2012; Lim et al., 2014, 2015), and there is also increasing evidence for alterations in the striatum, including the caudate and putamen (Cohen et al., 2006; Dannlowski et al., 2012; Teicher, 2015; Frodl et al., 2017).

Previous fMRI findings in this sample suggest that childhood abuse is associated with abnormally increased activation during error monitoring, in classical dorsomedial frontal error processing regions, such as the ACC and SMA (Lim et al., 2015). Other studies have also revealed increased activation during error monitoring in maltreated individuals in widespread regions, including the inferior parietal lobule, superior occipital lobe, thalamus, insula, putamen and midbrain (Bruce et al., 2013; Jankowski et al., 2017). Diminished functional connectivity for maltreated adolescents, relative to healthy controls, between the striatum and ACC and between SMA and IFC/DLPFC/DMPFC may contribute to the previously observed hyperactivity in error processing regions. For example, decreased fronto-cingulo-striatal connectivity could result in reduced inhibitory control of error monitoring networks, such as the ACC, resulting in increased activity of error processing regions and sensitivity to errors. We speculate that the increased sensitivity to errors as expressed in slower post-error reaction time and reduced fronto-cingulo-striatal connectivity in the abused adolescents relative to healthy controls could possibly be due to the constant need to monitor their actions to avoid potential painful mistakes. This hypothesis would be in line with evidence that environmental adversity such as punitive parental behaviors are associated with enhanced error-related negativity, which is localized to the medial frontal/ACC, in ERP studies (Gehring et al., 1993), and is related to hypersensitivity to punishment, hypervigilance (Santesso et al., 2011) and typical comorbidities of childhood maltreatment such as depression and anxiety (Olvet and Hajcak, 2008). Thus, we postulate that persistent harsh punishments in childhood may have sensitized the abused child to errors and led to a less communicative, poorly controlled, and therefore overactive, error-monitoring system.

The abused group demonstrated normal inhibitory capacity and normal functional connectivity for the successful stop trials, which is consistent with previous performance (Carrion et al., 2008) and fMRI activation findings using the same stop-signal paradigm (Elton et al., 2014; Lim et al., 2015). Studies that report impaired inhibitory activation generally used different tasks such as go/no-go (Carrion et al., 2008) and stop-change tasks (Mueller et al., 2010) and recruited youths who experienced early deprivation (Mueller et al., 2010), Post-Traumatic Stress Disorder (PTSD) and childhood trauma including sexual abuse and witnessing violence (Carrion et al., 2008), which were not included in our study rendering the findings not directly comparable. The one study that reported reduced functional connectivity during inhibition recruited adults with a wide range of maltreatment type and severity and carried out correlational graph theory analyses, not direct group comparisons (Elton et al., 2014). It is therefore not appropriate to directly compare these findings and future studies are needed to further examine the integrity of inhibitory networks in youth exposed to different types of maltreatment.

The specificity of abnormality findings in both the performance and connectivity analyses is interesting. Maltreated individuals showed normal inhibitory capacity but had slowed responses to go trials as well as showing abnormally enhanced post-error slowing suggesting that they are over-worried or over-cautious about making mistakes.

The functional connectivity abnormalities between medial frontal regions such as SMA, DMPFC and ACC, and lateral prefrontal cortex and basal ganglia which form typical error monitoring networks, but the integrity of networks during successful stop trials, suggests that only error monitoring networks but not inhibitory networks are abnormal in this population. Medial prefrontal regions, in particular the DMPFC, ACC and SMA, putamen, and bilateral dorsolateral and inferior prefrontal regions form a typical error monitoring network (Rubia et al., 2007, 2011; Li et al., 2008), while right IFC, caudate, and pre-SMA have more commonly been associated with successful inhibitory control in the same and similar tasks in children and adults (Rubia et al., 2003, 2007, 2013; Rae et al., 2014). While there is overlap in some regions (SMA, IFC, caudate), potentially due to the fact that failed inhibitions are attempted inhibitions that are too slow to succeed, there is evidence for differential network implications for inhibitory control and error monitoring (Hochman et al., 2015; Iannaccone et al., 2015). It thus seems that in adolescents who were subjected to physical maltreatment in childhood, specific networks involving error monitoring have maladapted, presumably due to a lifetime of harsh consequences to mistakes, while related inhibitory right IFC-caudate-pre-SMA networks are unaffected, suggesting dissociated maltreatment-related developmental abnormalities in related and partially overlapping networks.

We observed no correlations between age and functional connectivity networks in either controls nor people with maltreatment. The findings are not in line with evidence for developmental changes in functional connectivity networks with age (Rubia, 2013; Allard and Kensinger, 2014).

Only 2 of our 10 seed regions showed significant findings. This may possibly be caused by type 1 errors resulting from the use of a stringent FWER corrected cluster threshold. The fact that no connectivity differences were observed for the IFC seed region in our study compared to that of Elton et al. (2014) may reflect the fact that this region is more typically associated with inhibition than error processing (Rubia et al., 2003; Aron et al., 2014) and the abused group in the current study demonstrated normal inhibitory capacity. It may also reflect differences in paradigm, age or maltreatment type as discussed above. Fronto-cingulo-striatal performance monitoring networks have been shown to still mature between childhood and adulthood (Rubia et al., 2007; Velanova et al., 2008; Stevens et al., 2009). This protracted development may render these networks more vulnerable to environmental stressors such as child abuse than other neural networks.

Our preliminary GxE findings in right cingulo-striatal connectivity are intriguing as they suggest that connectivity deficits in these stress-susceptible error processing brain networks were influenced by the abuse experience and possibly exacerbated in the presence of the *MAOA-Low* genotype. *MAOA-Low* carriers exposed to childhood maltreatment have been shown to demonstrate higher impulsivity (Huang et al., 2004) and increased risks for mood and antisocial behavioral disorders (Caspi et al., 2002; Viding and Frith, 2006; Taylor and Kim-Cohen, 2007; Weder et al., 2009) than their *MAOA-High* counterparts. The *MAOA-Low* genotype has also been related to hyper-responsivity of the brain’s threat detection and emotion regulation circuits (Viding and Frith, 2006). The current results may extend this to error monitoring networks and the combined effect of constantly monitoring for potential threat and errors in abusive settings may contribute to the observed reduction in functional connectivity. No group-by-5-*HTTLPR* effect was observed suggesting that the specific cingulo-striatal functional connectivity deficits observed during error processing are not modulated by *5-HTTLPR* genotype.

Among the strengths of the current study is that all participants were medication-naïve, drug-free and the abuse experience was carefully assessed and corroborated by social service records. Furthermore the abuse severity in our participants was relatively high compared to previous studies. It is unclear to what extent pubertal development, malnutrition, prenatal drug exposure and presence of current life stressors may have influenced the findings. The SES measure used is limited, as it does not provide information on parents’ income and education; however, youth often have difficulties in reporting this information (Currie et al., 1997). Although we recruited participants exposed to childhood physical abuse, it is unrealistic to separate physical abuse from typically co-occurring emotional abuse and neglect (Edwards et al., 2003; Trickett et al., 2011); hence, our abuse group had experienced emotional abuse and neglect as well. Another limitation is the inclusion of mixed genders as maltreatment may affect the genders differently (Cooke and Weathington, 2014). Given that several of the participants would have been in the midst of puberty, the lack of pubertal information is a limitation as pubertal development could have influenced the findings.

The sample size is very small for studying genotype effects and all genotype analyses should therefore be considered underpowered and explorative. In addition, current height and extent thresholds may not accommodate the influence of sample size nor non-gaussian distributions for auto-correlated measurements (Woo et al., 2014).

In summary, using medication-naïve, drug-free, carefully assessed age-matched groups of young people exposed to severe childhood maltreatment and healthy controls, we found that abused participants had reduced functional connectivity between right putamen and bilateral caudate, ACC and left putamen and between right SMA and IFC and DLPFC/DMPFC regions during unsuccessful inhibition/error monitoring, but showed no abnormal inhibitory connectivity. Furthermore connectivity deficits were associated with longer abuse duration and moderated by *MAOA* genotype. Hence, in response to an environment where errors frequently predict the occurrence of abuse, maltreated individuals may develop a reduction in communication between brain regions involved in error processing resulting in a greater sensitivity to errors. These findings represent a first step towards the delineation of neurofunctional connectivity abnormalities associated with child abuse, which hopefully may ultimately lead to the development of specific treatment strategies for victims of childhood maltreatment.

## Author Contributions

KR, MAM, KM, AS and HH were involved with the conception and design of the research. Data collection and analysis was carried out by HH and LL under the supervision of KR. Genotyping analysis was carried out by CC, XX and GB. HH was responsible for drafting the article, which was critically revised by LL, KR and MAM. Final approval of the version to be published was given by all co-authors.

## Conflict of Interest Statement

KR has received speaker’s honoraria from Lilly and Shire and received a grant from Lilly for another project. MAM has acted as a consultant for Cambridge Cognition, Lundbeck and Quintiles and has received fees from Shire for contribution towards education. KM has received research and educational grants from Glaxo Smith Kline and Shire pharmaceuticals and has served on the advisory boards of Janssen, Eli Lily and Shire pharmaceuticals. KM has also received honoraria for speaking at conferences organized by Janssen, Eli Lilly and Shire pharmaceuticals. The other authors declare that the research was conducted in the absence of any commercial or financial relationships that could be construed as a potential conflict of interest.

## Footnotes

^1^http://www.testfield.co.uk

^2^www.fil.ion.ucl.ac.uk/spm

^3^http://www.nitrc.org/projects/gppi

## References

[B1] Ackerman P. T. Newton J. E. Mcpherson W. B. Jones J. G. Dykman R. A. (1998). Prevalence of post traumatic stress disorder and other psychiatric diagnoses in three groups of abused children (sexual, physical and both). Child Abuse Negl. 22, 759–774. 9717613 10.1016/s0145-2134(98)00062-3

[B2] Allard E. S. Kensinger E. A. (2014). Age-related differences in functional connectivity during cognitive emotion regulation. J. Gerontol. B Psychol. Sci. Soc. Sci. 69, 852–860. 10.1093/geronb/gbu108 25209373 PMC4296140

[B3] Aron A. R. Robbins T. W. Poldrack R. A. (2014). Inhibition and the right inferior frontal cortex: one decade on. Trends Cogn. Sci. 18, 177–185. 10.1016/j.tics.2013.12.003 24440116

[B4] Beckett C. Castle J. Rutter M. Sonuga-Barke E. J. (2010). VI. Institutional deprivation, specific cognitive functions, and scholastic achievement: English and Romanian adoptee (ERA) study findings. Monogr. Soc. Res. Child Dev. 75, 125–142. 10.1111/j.1540-5834.2010.00553.x 20500636

[B5] Bernstein D. Fink L. (1998). Childhood Trauma Questionnaire: A retrospective Self-report. Manual. San Antonio, TX: The Psychological Cooperation.

[B6] Bifulco A. Brown G. W. Harris T. O. (1994). Childhood experience of care and abuse (CECA): a retrospective interview measure. J. Child Psychol. Psychiatry 35, 1419–1435. 10.1111/j.1469-7610.1994.tb01284.x 7868637

[B93] Bluhm R. L. Williamson P. C. Osuch E. A. Frewen P. A. Stevens T. K. Boksman K. (2009). Alterations in default network connectivity in posttraumatic stress disorder related to early-life trauma. J. Psychiatry Neurosci. 34, 187–194. 19448848 PMC2674971

[B7] Bruce J. Fisher P. A. Graham A. M. Moore W. E. Peake S. J. Mannering A. M. (2013). Patterns of brain activation in foster children and nonmaltreated children during an inhibitory control task. Dev. Psychopathol. 25, 931–941. 10.1017/s095457941300028x 24229540 PMC3831359

[B8] Carrion V. G. Garrett A. Menon V. Weems C. F. Reiss A. L. (2008). Posttraumatic stress symptoms and brain function during a response-inhibition task: an fMRI study in youth. Depress. Anxiety 25, 514–526. 10.1002/da.20346 17598145

[B9] Caspi A. McClay J. Moffitt T. E. Mill J. Martin J. Craig I. W.. (2002). Role of genotype in the cycle of violence in maltreated children. Science 297, 851–854. 10.1126/science.1072290 12161658

[B10] Charney D. S. (2004). Psychobiological mechanisms of resilience and vulnerability: implications for successful adaptation to extreme stress. Focus 2, 368–391. 10.1176/foc.2.3.368 14754765

[B11] Chipman P. Jorm A. F. Prior M. Sanson A. Smart D. Tan X.. (2007). No interaction between the serotonin transporter polymorphism (5-HTTLPR) and childhood adversity or recent stressful life events on symptoms of depression: results from two community surveys. Am. J. Med. Genet. B Neuropsychiatr. Genet. 144B, 561–565. 10.1002/ajmg.b.30480 17450557

[B12] Choi J. Jeong B. Rohan M. L. Polcari A. M. Teicher M. H. (2009). Preliminary evidence for white matter tract abnormalities in young adults exposed to parental verbal abuse. Biol. Psychiatry 65, 227–234. 10.1016/j.biopsych.2008.06.022 18692174 PMC2652864

[B13] Chorbov V. M. Lobos E. A. Todorov A. A. Heath A. C. Botteron K. N. Todd R. D. (2007). Relationship of 5-HTTLPR genotypes and depression risk in the presence of trauma in a female twin sample. Am. J. Med. Genet. B Neuropsychiatr. Genet. 144B, 830–833. 10.1002/ajmg.b.30534 17455215

[B14] Cicchetti D. Rogosch F. A. Sturge-Apple M. L. (2007). Interactions of child maltreatment and serotonin transporter and monoamine oxidase a polymorphisms: depressive symptomatology among adolescents from low socioeconomic status backgrounds. Dev. Psychopathol. 19, 1161–1180. 10.1017/s0954579407000600 17931441

[B15] Cohen R. A. Grieve S. Hoth K. F. Paul R. H. Sweet L. Tate D.. (2006). Early life stress and morphometry of the adult anterior cingulate cortex and caudate nuclei. Biol. Psychiatry 59, 975–982. 10.1016/j.biopsych.2005.12.016 16616722

[B16] Cooke B. M. Weathington J. M. (2014). Human and animal research into sex-specific effects of child abuse. Horm. Behav. 65, 416–426. 10.1016/j.yhbeh.2014.03.004 24657521

[B18] Currie C. E. Elton R. A. Todd J. Platt S. (1997). Indicators of socioeconomic status for adolescents: the WHO health behaviour in school-aged children survey. Health Educ. Res. 12, 385–397. 10.1093/her/12.3.385 10174221

[B17] Currie C. Molcho M. Boyce W. Holstein B. R. Torsheim T. R. Richter M. (2008). Researching health inequalities in adolescents: the development of the health behaviour in school-aged children (HBSC) family affluence scale. Soc. Sci. Med. 66, 1429–1436. 10.1016/j.socscimed.2007.11.024 18179852

[B19] Damoiseaux J. S. Greicius M. D. (2009). Greater than the sum of its parts: a review of studies combining structural connectivity and resting-state functional connectivity. Brain Struct. Funct. 213, 525–533. 10.1007/s00429-009-0208-6 19565262

[B20] Dannlowski U. Stuhrmann A. Beutelmann V. Zwanzger P. Lenzen T. Grotegerd D.. (2012). Limbic scars: long-term consequences of childhood maltreatment revealed by functional and structural magnetic resonance imaging. Biol. Psychiatry 71, 286–293. 10.1016/j.biopsych.2011.10.021 22112927

[B21] De Bellis M. D. Hooper S. R. Spratt E. G. Woolley D. P. (2009). Neuropsychological findings in childhood neglect and their relationships to pediatric PTSD. J. Int. Neuropsychol. Soc. 15, 868–878. 10.1017/s1355617709990464 19703321 PMC3036972

[B22] Deckert J. Catalano M. Syagailo Y. V. Bosi M. Okladnova O. Di Bella D.. (1999). Excess of high activity monoamine oxidase a gene promoter alleles in female patients with panic disorder. Hum. Mol. Genet. 8, 621–624. 10.1093/hmg/8.4.621 10072430

[B23] Dennis M. Francis D. J. Cirino P. T. Schachar R. Barnes M. A. Fletcher J. M. (2009). Why IQ is not a covariate in cognitive studies of neurodevelopmental disorders. J. Int. Neuropsychol. Soc. 15, 331–343. 10.1017/s1355617709090481 19402919 PMC3075072

[B24] Deprince A. P. Weinzierl K. M. Combs M. D. (2009). Executive function performance and trauma exposure in a community sample of children. Child Abuse Negl. 33, 353–361. 10.1016/j.chiabu.2008.08.002 19477515

[B25] Edwards V. J. Holden G. W. Felitti V. J. Anda R. F. (2003). Relationship between multiple forms of childhood maltreatment and adult mental health in community respondents: results from the adverse childhood experiences study. Am. J. Psychiatry 160, 1453–1460. 10.1176/appi.ajp.160.8.1453 12900308

[B26] El Hage W. Powell J. Surguladze S. (2009). Vulnerability to depression: what is the role of stress genes in gene× environment interaction? Psychol. Med. 39, 1407–1411. 10.1017/s0033291709005236 19215634

[B27] Elton A. Tripathi S. P. Mletzko T. Young J. Cisler J. M. James G. A.. (2014). Childhood maltreatment is associated with a sex-dependent functional reorganization of a brain inhibitory control network. Hum. Brain Mapp. 35, 1654–1667. 10.1002/hbm.22280 23616424 PMC3779516

[B28] Eluvathingal T. J. Chugani H. T. Behen M. E. Juhász C. Muzik O. Maqbool M.. (2006). Abnormal brain connectivity in children after early severe socioemotional deprivation: a diffusion tensor imaging study. Pediatrics 117, 2093–2100. 10.1542/peds.2005-1727 16740852

[B29] Fonzo G. A. Flagan T. M. Sullivan S. Allard C. B. Grimes E. M. Simmons A. N.. (2013). Neural functional and structural correlates of childhood maltreatment in women with intimate-partner violence-related posttraumatic stress disorder. Psychiatry Res. 211, 93–103. 10.1016/j.pscychresns.2012.08.006 23154098 PMC3570713

[B30] Frodl T. Janowitz D. Schmaal L. Tozzi L. Dobrowolny H. Stein D. J.. (2017). Childhood adversity impacts on brain subcortical structures relevant to depression. J. Psychiatr. Res. 86, 58–65. 10.1016/j.jpsychires.2016.11.010 27918926 PMC5564511

[B31] Gehring W. J. Goss B. Coles M. G. Meyer D. E. Donchin E. (1993). A neural system for error detection and compensation. Psychol. Sci. 4, 385–390. 10.1111/j.1467-9280.1993.tb00586.x

[B32] Goodman R. Ford T. Richards H. Gatward R. Meltzer H. (2000). The development and well-being assessment: description and initial validation of an integrated assessment of child and adolescent psychopathology. J. Child Psychol. Psychiatry 41, 645–655. 10.1111/j.1469-7610.2000.tb02345.x 10946756

[B34] Hart H. Chantiluke K. Cubillo A. I. Smith A. B. Simmons A. Brammer M. J.. (2014). Pattern classification of response inhibition in ADHD: toward the development of neurobiological markers for ADHD. Hum. Brain Mapp. 35, 3083–3094. 10.1002/hbm.22386 24123508 PMC4190683

[B35] Hart H. Lim L. Mehta M. A. Simmons A. Mirza K. Rubia K. (in press). Altered fear processing in adolescents with a history of severe childhood maltreatment: an fMRI study. Psychol. Med. 10.1017/S0033291716003585 PMC6088776 29429419

[B33] Hart H. Rubia K. (2012). Neuroimaging of child abuse: a critical review. Front. Hum. Neurosci. 6:52. 10.3389/fnhum.2012.00052 22457645 PMC3307045

[B36] Heim C. M. Mayberg H. S. Mletzko T. Nemeroff C. B. Pruessner J. C. (2013). Decreased cortical representation of genital somatosensory field after childhood sexual abuse. Am. J. Psychiatry 170, 616–623. 10.1176/appi.ajp.2013.12070950 23732967

[B37] Hochman E. Y. Wang S. Milner T. E. Fellows L. K. (2015). Double dissociation of error inhibition and correction deficits after basal ganglia or dorsomedial frontal damage in humans. Neuropsychologia 69, 130–139. 10.1016/j.neuropsychologia.2015.01.023 25600344

[B38] Holmes A. (2008). Genetic variation in cortico-amygdala serotonin function and risk for stress-related disease. Neurosci. Biobehav. Rev. 32, 1293–1314. 10.1016/j.neubiorev.2008.03.006 18439676 PMC2561331

[B39] Huang Y.-Y. Cate S. P. Battistuzzi C. Oquendo M. A. Brent D. Mann J. J. (2004). An association between a functional polymorphism in the monoamine oxidase a gene promoter, impulsive traits and early abuse experiences. Neuropsychopharmacology 29, 1498–1505. 10.1038/sj.npp.1300455 15150530

[B40] Iannaccone R. Hauser T. U. Staempfli P. Walitza S. Brandeis D. Brem S. (2015). Conflict monitoring and error processing: new insights from simultaneous EEG-fMRI. Neuroimage 105, 395–407. 10.1016/j.neuroimage.2014.10.028 25462691

[B41] Jankowski K. F. Bruce J. Beauchamp K. G. Roos L. E. Moore W. E. Fisher P. A. (2017). Preliminary evidence of the impact of early childhood maltreatment and a preventive intervention on neural patterns of response inhibition in early adolescence. Dev. Sci. 20:e12413. 10.1111/desc.12413 27061609 PMC5055407

[B42] Jedd K. Hunt R. H. Cicchetti D. Hunt E. Cowell R. A. Rogosch F. A.. (2015). Long-term consequences of childhood maltreatment: altered amygdala functional connectivity. Dev. Psychopathol. 27, 1577–1589. 10.1017/s0954579415000954 26535945 PMC4635964

[B43] Kim-Cohen J. Caspi A. Taylor A. Williams B. Newcombe R. Craig I. W.. (2006). MAOA, maltreatment, and gene-environment interaction predicting children’s mental health: new evidence and a meta-analysis. Mol. Psychiatry 11, 903–913. 10.1038/sj.mp.4001851 16801953

[B44] Laucht M. Treutlein J. Blomeyer D. Buchmann A. F. Schmid B. Becker K.. (2009). Interaction between the 5-HTTLPR serotonin transporter polymorphism and environmental adversity for mood and anxiety psychopathology: evidence from a high-risk community sample of young adults. Int. J. Neuropsychopharmacol. 12, 737–747. 10.1017/s1461145708009875 19154632

[B45] Li C. S. R. Huang C. Yan P. S. Paliwal P. Constable R. T. Sinha R. (2008). Neural correlates of post-error slowing during a stop signal task: a functional magnetic resonance imaging study. J. Cogn. Neurosci. 20, 1021–1029. 10.1162/jocn.2008.20071 18211230 PMC2597347

[B46] Lim L. Hart H. Mehta M. A. Simmons A. Mirza K. Rubia K. (2015). Neural correlates of error processing in young people with a history of severe childhood abuse: an fMRI study. Am. J. Psychiatry 172, 892–900. 10.1176/appi.ajp.2015.14081042 25882324

[B47] Lim L. Radua J. Rubia K. (2014). Gray matter abnormalities in childhood maltreatment: a voxel-wise meta-analysis. Am. J. Psychiatry 171, 854–863. 10.1176/appi.ajp.2014.13101427 24781447

[B48] Maldjian J. A. Laurienti P. J. Kraft R. A. Burdette J. H. (2003). An automated method for neuroanatomic and cytoarchitectonic atlas-based interrogation of fMRI data sets. Neuroimage 19, 1233–1239. 10.1016/s1053-8119(03)00169-1 12880848

[B49] Matthews J. N. Altman D. G. (1996). Statistics notes: interaction 2: compare effect sizes not P values. BMJ 313:808. 10.1136/bmj.313.7060.808 8842080 PMC2352195

[B50] McLaren D. G. Ries M. L. Xu G. Johnson S. C. (2012). A generalized form of context-dependent psychophysiological interactions (gPPI): a comparison to standard approaches. Neuroimage 61, 1277–1286. 10.1016/j.neuroimage.2012.03.068 22484411 PMC3376181

[B51] Meyer A. Proudfit G. H. Bufferd S. J. Kujawa A. J. Laptook R. S. Torpey D. C.. (2015). Self-reported and observed punitive parenting prospectively predicts increased error-related brain activity in six-year-old children. J. Abnorm. Child Psychol. 43, 821–829. 10.1007/s10802-014-9918-1 25092483 PMC5302091

[B52] Meyer-Lindenberg A. Buckholtz J. W. Kolachana B. Hariri A. R. Pezawas L. Blasi G.. (2006). Neural mechanisms of genetic risk for impulsivity and violence in humans. Proc. Natl. Acad. Sci. U S A 103, 6269–6274. 10.1073/pnas.0511311103 16569698 PMC1458867

[B53] Mezzacappa E. Kindlon D. Earls F. (2001). Child abuse and performance task assessments of executive functions in boys. J. Child Psychol. Psychiatry 42, 1041–1048. 10.1111/1469-7610.00803 11806686

[B54] Miller G. Chapman J. (2001). Misunderstanding analysis of covariance. J. Abnorm. Psychol. 110, 40–48. 10.1037//0021-843x.110.1.40 11261398

[B55] Mueller S. C. Maheu F. S. Dozier M. Peloso E. Mandell D. Leibenluft E.. (2010). Early-life stress is associated with impairment in cognitive control in adolescence: an fMRI study. Neuropsychologia 48, 3037–3044. 10.1016/j.neuropsychologia.2010.06.013 20561537 PMC2916226

[B56] Nachev P. Kennard C. Husain M. (2008). Functional role of the supplementary and pre-supplementary motor areas. Nat. Rev. Neurosci. 9, 856–869. 10.1038/nrn2478 18843271

[B57] Navalta C. P. Polcari A. Webster D. M. Boghossian A. Teicher M. H. (2006). Effects of childhood sexual abuse on neuropsychological and cognitive function in college women. J. Neuropsychiatry Clin. Neurosci. 18, 45–53. 10.1176/appi.neuropsych.18.1.45 16525070

[B58] Nugent N. R. Tyrka A. R. Carpenter L. L. Price L. H. (2011). Gene-environment interactions: early life stress and risk for depressive and anxiety disorders. Psychopharmacology 214, 175–196. 10.1007/s00213-010-2151-x 21225419 PMC3615637

[B59] Olsson C. Byrnes G. Lotfi-Miri M. Collins V. Williamson R. Patton C.. (2005). Association between 5-HTTLPR genotypes and persisting patterns of anxiety and alcohol use: results from a 10-year longitudinal study of adolescent mental health. Mol. Psychiatry 10, 868–876. 10.1038/sj.mp.4001677 15852063

[B60] Olvet D. M. Hajcak G. (2008). The error-related negativity (ERN) and psychopathology: toward an endophenotype. Clin. Psychol. Rev. 28, 1343–1354. 10.1016/j.cpr.2008.07.003 18694617 PMC2615243

[B61] Paul R. Henry L. Grieve S. M. Guilmette T. J. Niaura R. Bryant R.. (2008). The relationship between early life stress and microstructural integrity of the corpus callosum in a non-clinical population. Neuropsychiatr. Dis. Treat. 4, 193–201. 10.2147/ndt.s1549 18728817 PMC2515911

[B62] Pechtel P. Pizzagalli D. A. (2011). Effects of early life stress on cognitive and affective function: an integrated review of human literature. Psychopharmacology (Berl) 214, 55–70. 10.1007/s00213-010-2009-2 20865251 PMC3050094

[B63] Pollak S. D. Nelson C. A. Schlaak M. F. Roeber B. J. Wewerka S. S. Wiik K. L.. (2010). Neurodevelopmental effects of early deprivation in postinstitutionalized children. Child Dev. 81, 224–236. 10.1111/j.1467-8624.2009.01391.x 20331664 PMC2846096

[B64] Power J. D. Barnes K. A. Snyder A. Z. Schlaggar B. L. Petersen S. E. (2012). Spurious but systematic correlations in functional connectivity MRI networks arise from subject motion. Neuroimage 59, 2142–2154. 10.1016/j.neuroimage.2011.10.018 22019881 PMC3254728

[B65] Radford L. Corral S. Bradley C. Fisher H. L. (2013). The prevalence and impact of child maltreatment and other types of victimization in the UK: findings from a population survey of caregivers, children and young people and young adults. Child Abuse Negl. 37, 801–813. 10.1016/j.chiabu.2013.02.004 23522961

[B66] Rae C. L. Hughes L. E. Weaver C. Anderson M. C. Rowe J. B. (2014). Selection and stopping in voluntary action: a meta-analysis and combined fMRI study. Neuroimage 86, 381–391. 10.1016/j.neuroimage.2013.10.012 24128740 PMC3898966

[B67] Rubia K. (2013). Functional brain imaging across development. Eur. Child Adolesc. Psychiatry 22, 719–731. 10.1007/s00787-012-0291-8 22729957 PMC3853580

[B68] Rubia K. Halari R. Mohammad A. M. Taylor E. Brammer M. (2011). Methylphenidate normalizes frontocingulate underactivation during error processing in attention-deficit/hyperactivity disorder. Biol. Psychiatry 70, 255–262. 10.1016/j.biopsych.2011.04.018 21664605 PMC3139835

[B69] Rubia K. Lim L. Ecker C. Halari R. Giampietro V. Simmons A.. (2013). Effects of age and gender on neural networks of motor response inhibition: from adolescence to mid-adulthood. Neuroimage 83, 690–703. 10.1016/j.neuroimage.2013.06.078 23845427

[B70] Rubia K. Russell T. Overmeyer S. Brammer M. J. Bullmore E. T. Sharma T.. (2001). Mapping motor inhibition: conjunctive brain activations across different versions of go/no-go and stop tasks. Neuroimage 13, 250–261. 10.1006/nimg.2000.0685 11162266

[B71] Rubia K. Smith A. B. Brammer M. J. Taylor E. (2003). Right inferior prefrontal cortex mediates response inhibition while mesial prefrontal cortex is responsible for error detection. Neuroimage 20, 351–358. 10.1016/s1053-8119(03)00275-1 14527595

[B72] Rubia K. Smith A. B. Taylor E. Brammer M. (2007). Linear age-correlated functional development of right inferior fronto-striato-cerebellar networks during response inhibition and anterior cingulate during error-related processes. Hum. Brain Mapp. 28, 1163–1177. 10.1002/hbm.20347 17538951 PMC6871440

[B74] Santesso D. L. Dzyundzyak A. Segalowitz S. J. (2011). Age, sex and individual differences in punishment sensitivity: factors influencing the feedback-related negativity. Psychophysiology 48, 1481–1489. 10.1111/j.1469-8986.2011.01229.x 21711354

[B75] Shih J. Chen K. Ridd M. (1999). Monoamine oxidase: from genes to behavior. Annu. Rev. Neurosci. 22, 197–217. 10.1146/annurev.neuro.22.1.197 10202537 PMC2844879

[B76] Siegel J. S. Power J. D. Dubis J. W. Vogel A. C. Church J. A. Schlaggar B. L.. (2014). Statistical improvements in functional magnetic resonance imaging analyses produced by censoring high-motion data points. Hum. Brain Mapp. 35, 1981–1996. 10.1002/hbm.22307 23861343 PMC3895106

[B77] Simmonds D. J. Pekar J. J. Mostofsky S. H. (2008). Meta-analysis of Go/No-go tasks, demonstrating that fMRI activation associated with response inhibition is task-dependent. Neuropsychologia 46, 224–232. 10.1016/j.neuropsychologia.2007.07.015 17850833 PMC2327217

[B78] Simmons A. Moore E. Williams S. C. R. (1999). Quality control for functional magnetic resonance imaging using automated data analysis and shewhart charting. Magn. Reson. Med. 41, 1274–1278. 10.1002/(sici)1522-2594(199906)41:6<1274::aid-mrm27>3.3.co;2-t 10371463

[B79] Stevens M. C. Kiehl K. A. Pearlson G. D. Calhoun V. D. (2009). Brain network dynamics during error commission. Hum. Brain Mapp. 30, 24–37. 10.1002/hbm.20478 17979124 PMC2669663

[B80] Surtees P. G. Wainwright N. W. Willis-Owen S. A. Luben R. Day N. E. Flint J. (2006). Social adversity, the serotonin transporter (5-HTTLPR) polymorphism and major depressive disorder. Biol. Psychiatry 59, 224–229. 10.1016/j.biopsych.2005.07.014 16154545

[B81] Swick D. Ashley V. Turken U. (2008). Left inferior frontal gyrus is critical for response inhibition. BMC Neurosci. 9:102. 10.1186/1471-2202-9-102 18939997 PMC2588614

[B82] Taylor A. Kim-Cohen J. (2007). Meta-analysis of gene-environment interactions in developmental psychopathology. Dev. Psychopathol. 19, 1029–1037. 10.1017/s095457940700051x 17931432

[B83] Teicher M. H. (2015). “Gender-specific influence of type and timing of childhood maltreatment on caudate, putamen and nucleus accumbens volume,” in Proceedings of the 70th Annual Scientific Meeting of the Society-of-Biological-Psychiatry (Toronto, ON).

[B84] Teicher M. H. Andersen S. L. Polcari A. Anderson C. M. Navalta C. P. Kim D. M. (2003). The neurobiological consequences of early stress and childhood maltreatment. Neurosci. Biobehav. Rev. 27, 33–44. 10.1016/s0149-7634(03)00007-1 12732221

[B85] Trickett P. K. Kim K. Prindle J. (2011). Variations in emotional abuse experiences among multiply maltreated young adolescents and relations with developmental outcomes. Child Abuse Negl. 35, 876–886. 10.1016/j.chiabu.2011.08.001 22018516 PMC3221462

[B86] Tzourio-Mazoyer N. Landeau B. Papathanassiou D. Crivello F. Etard O. Delcroix N.. (2002). Automated anatomical labeling of activations in SPM using a macroscopic anatomical parcellation of the MNI MRI single-subject brain. Neuroimage 15, 273–289. 10.1006/nimg.2001.0978 11771995

[B87] Van der Werff S. Pannekoek J. Veer I. van Tol M.-J. Aleman A. Veltman D.. (2013). Resting-state functional connectivity in adults with childhood emotional maltreatment. Psychol. Med. 43, 1825–1836. 10.1017/s0033291712002942 23254143

[B88] Van Dijk K. R. A. Sabuncu M. R. Buckner R. L. (2012). The influence of head motion on intrinsic functional connectivity MRI. Neuroimage 59, 431–438. 10.1016/j.neuroimage.2011.07.044 21810475 PMC3683830

[B89] Velanova K. Wheeler M. E. Luna B. (2008). Maturational changes in anterior cingulate and frontoparietal recruitment support the development of error processing and inhibitory control. Cereb. Cortex 18, 2505–2522. 10.1093/cercor/bhn012 18281300 PMC2733315

[B90] Viding E. Frith U. (2006). Genes for susceptibility to violence lurk in the brain. Proc. Natl. Acad. Sci. U S A 103, 6085–6086. 10.1073/pnas.0601350103 16606856 PMC1458834

[B91] Wechsler D. (1999). Wechsler Abbreviated Scale of Intelligence. San Antonio, TX: The Psychological Corporation.

[B92] Weder N. Yang B. Z. Douglas-Palumberi H. Massey J. Krystal J. H. Gelernter J.. (2009). MAOA genotype, maltreatment, and aggressive behavior: the changing impact of genotype at varying levels of trauma. Biol. Psychiatry 65, 417–424. 10.1016/j.biopsych.2008.09.013 18996506 PMC3816252

[B94] Woo C.-W. Krishnan A. Wager T. D. (2014). Cluster-extent based thresholding in fMRI analyses: pitfalls and recommendations. . Neuroimage 91, 412–419. 10.1016/j.neuroimage.2013.12.058 24412399 PMC4214144

[B95] Zoroğlu S. Erdal M. E. Alaşehirli B. Erdal N. Sivasli E. Tutkun H.. (2002). Significance of serotonin transporter gene 5-HTTLPR and variable number of tandem repeat polymorphism in attention deficit hyperactivity disorder. Neuropsychobiology 45, 176–181. 10.1159/000063667 12097805

